# Involvement of the nuclear factor-κB transcriptional complex in prefrontal cortex immune activation in bipolar disorder

**DOI:** 10.1038/s41398-020-01092-x

**Published:** 2021-01-12

**Authors:** Kaitlyn M. Roman, Aaron K. Jenkins, David A. Lewis, David W. Volk

**Affiliations:** 1 Department of Psychiatry, University of Pittsburgh, Pittsburgh, PA 15213 USA; 2 Department of Neuroscience, University of Pittsburgh, Pittsburgh, PA 15213 USA; 3 Veterans Integrated Service Network 4 Mental Illness Research Education and Clinical Center (MIRECC), VA Pittsburgh Healthcare System, Pittsburgh, PA 15240 USA

**Keywords:** Schizophrenia, Molecular neuroscience

## Abstract

Bipolar disorder and schizophrenia have multiple clinical and genetic features in common, including shared risk associated with overlapping susceptibility loci in immune-related genes. Higher activity of the nuclear factor-κB (NF-κB) transcription factor complex, which regulates the transcription of multiple immune markers, has been reported to contribute to immune activation in the prefrontal cortex in schizophrenia. These findings suggest the hypothesis that elevated NF-κB activity is present in the prefrontal cortex in bipolar disorder in a manner similar to that seen in schizophrenia. Therefore, we quantified levels of NF-κB-related mRNAs in the prefrontal cortex of 35 matched pairs of bipolar disorder and unaffected comparison subjects using quantitative PCR. We found that transcript levels were higher in the prefrontal cortex of bipolar disorder subjects for several NF-κB family members, NF-κB activation receptors, and NF-κB-regulated mRNAs, and were lower for an NF-κB inhibitor. Transcript levels for NF-κB family members, NF-κB activation receptors, and NF-κB-regulated mRNAs levels were also highly correlated with each other. This pattern of elevated transcript levels for NF-κB-related markers in bipolar disorder is similar to that previously reported in schizophrenia, suggesting that cortical immune activation is a shared pathophysiological feature between the two disorders.

## Introduction

Bipolar disorder shares several clinical and genetic features with schizophrenia. For example, certain clinical features, including cognitive dysfunction and psychotic symptoms^1^, are present in both schizophrenia and bipolar disorder. In addition, schizophrenia and bipolar disorder share some genetic risk, including overlapping susceptibility loci in immune-related genes^2–5^.

**Fig. 1:**
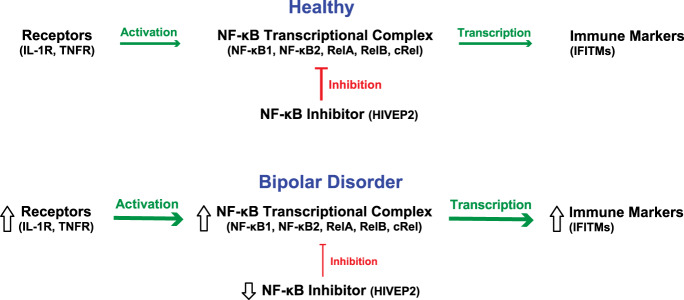
NF-κB activation pathway in bipolar disorder. The NF-κB transcriptional complex is composed of heterodimers between NF-κB family members, including NF-κB1, NF-κB2, and other members of the NF-κB family that contain Rel homology domains including RelA, RelB, and cRel. Receptor activation (IL-1R, TNFR) increases transcriptional activity (green arrow) of the NF-κB transcriptional complex, leading to higher mRNA levels for NF-κB-regulated immune markers, such as IFITMs. In contrast, the NF-κB site-binding protein HIVEP2 inhibits NF-κB transcriptional activity (red line). Transcript levels in bipolar disorder subjects relative to unaffected comparison subjects are indicated by the direction of the open arrows.

Recent studies have uncovered molecular evidence indicating the presence of cortical immune activation in individuals with schizophrenia. For example, markedly elevated transcript levels for multiple immune markers, including interferon-induced transmembrane proteins (IFITMs), have been consistently reported in the prefrontal cortex (PFC) in schizophrenia^6–9^. The nuclear factor-κB (NF-κB) transcription factor complex, which is comprised of heterodimers between NF-κB family members (i.e., NF-κB1, NF-κB2, RelA, RelB, and cRel), provides direct transcriptional regulation over the expression of multiple immune markers including IFITMs (Fig. 1)^10–12^. Activity of the NF-κB complex is initiated by activation of certain receptors (e.g., IL-1R, TNFR) and is reduced by inhibitors that block binding of the NF-κB complex to DNA (e.g., HIVEP2). In schizophrenia, elevated mRNA levels for most NF-κB family members and activation receptors and lower mRNA levels for NF-κB inhibitors in the PFC have been reported^13–15^. Consequently, elevated NF-κB activity appears to play a central role in cortical immune activation in schizophrenia. These findings raise the question of whether the elevated NF-κB activity seen in schizophrenia is also present in bipolar disorder.

Therefore, informed by the results of our previous study of NF-κB-related mRNAs in schizophrenia subjects^13,14^, we utilized the same quantitative PCR approach to selectively examine levels for NF-κB-related mRNAs in the canonical pathway in bipolar disorder subjects. We tested the hypotheses that in the PFC of subjects with bipolar disorder, transcript levels are (1) higher for NF-κB family members and activation receptors, (2) higher for immune markers that are regulated by NF-κB activity (i.e. IFITMs) and (3) lower for an NF-κB inhibitor (i.e. HIVEP2). We also determined whether elevated NF-κB-related mRNAs were primarily associated with psychosis in bipolar disorder or subtypes of bipolar disorder (i.e. bipolar I disorder or other types of bipolar disorder).

## Materials and methods

### Human subjects

All procedures were approved by the University of Pittsburgh’s Committee for the Oversight of Research and Clinical Training Involving Decedents and Institutional Review Board for Biomedical Research. Brain specimens were obtained during routine autopsies conducted at the Allegheny County Medical Examiner’s Office after consent was obtained from next-of-kin. Brain tissue from all subjects underwent a detailed neuropathology assessment by a neuropathologist in the University of Pittsburgh Division of Neuropathology to confirm the absence of significant findings that would indicate the presence of vascular pathology, ischemic and hemorrhagic lesions, traumatic brain injury, and neurodegenerative diseases such as Alzheimer’s disease and Parkinson’s disease. All subjects died out-of-hospital with minimal to no agonal state events and did not die from immune/inflammation-related causes of death. An independent committee of experienced research clinicians made consensus DSMIV^16^ diagnoses, or confirmed the absence of any lifetime psychiatric diagnosis, for each subject using structured interviews with family members and review of medical records^17^. To reduce biological variance between groups, subjects with bipolar disorder (*n* = 35) were matched individually to one unaffected comparison subject for sex and as closely as possible for age (Supplemental Table S1). To control for experimental variance, samples from paired subjects were processed together. The mean age, postmortem interval, RNA integrity number (RIN), brain pH, and freezer storage time did not differ between subject groups (*t*_(68)_ ≤ 0.96, *p* ≥ 0.34; Table 1).

**Table 1:** Summary of demographic and postmortem characteristics of human subjects.

Parameter	Unaffected comparison	Bipolar disorder (%)
*N*	35	35
Sex	20M/15F	20M/15F
Race	32W/3B	34W/1B
Age (years)	46.4 ± 12.7	45.5 ± 12.2
Postmortem interval (h)	19.1 ± 5.1	20.5 ± 7.0
Freezer storage time (months)	103.0 ± 49.8	109.9 ± 45.1
Brain pH	6.7 ± 0.3	6.6 ± 0.3
RNA integrity number	8.1 ± 0.6	8.0 ± 0.6
Medications at time of death^a^		
Antipsychotic	–	12 (34.3)
Antidepressant	–	22 (62.9)
Benzodiazepine/anticonvulsant	–	16 (45.7)
Lithium	–	3 (8.6)

The mean (±standard deviation) age, postmortem interval, RNA integrity number (RIN), brain pH, and tissue freezer storage time did not differ between bipolar disorder subjects and their matched unaffected comparison subjects (all *t*_(68)_ ≤ 0.96, *p* ≥ 0.34).

^a^For medications at time of death, the number and percentage (in parentheses) of subjects in each applicable category are provided.

### Quantitative PCR

Frozen tissue blocks containing the middle portion of the right superior frontal sulcus for each subject were confirmed to contain PFC area 9 using Nissl-stained, cryostat tissue sections^18^. The gray-white matter boundary of PFC area 9 in a tissue block from each subject was carefully scored with a scalpel blade where the gray matter was cut perpendicular to the pia matter and had uniform thickness and the gray-white matter boundary was easily delineated, which ensured that cortical layers were evenly represented in the tissue sample with minimal white matter contamination^19,20^. The scored gray matter region of the tissue block was then digitally photographed, and the number of tissue sections (40 µm) required to collect ~30 mm^3^ of gray matter was determined for each subject. The calculated number of required tissue sections for each subject was then cut by cryostat, and gray matter was separately collected into a tube containing TRIzol reagent in a manner consistent with excellent RNA preservation^19,20^. Standardized dilutions of total RNA for each subject were used to synthesize cDNA. Quantitative PCR (qPCR) was then performed using the comparative cycle threshold (CT) method with Power SYBR Green dye and the ViiA-7 Real-Time PCR System (Applied Biosystems) with the technician blinded to subject diagnosis until mRNA levels had been quantified for all subjects, as previously described^21^ (Supplemental Table S2). Three reference genes (beta actin, cyclophilin A, and GAPDH) were used to normalize target mRNA levels, as previously described^22^. The difference in CT (dCT) for each target transcript was calculated by subtracting the geometric mean CT for the three reference genes from the CT of the target transcript (mean of four replicate measures). Because dCT represents the log2-transformed expression ratio of each target transcript to the reference genes, the relative level of the target transcript for each subject is reported as 2^−dCT^ (refs. ^23,24^).

### Statistical analysis

Analyses of covariance (ANCOVA) were first conducted to determine whether mRNA levels were related to sex, age at death, postmortem interval, brain pH, RIN, and/or storage time. We found that HIVEP2 mRNA levels were related to age (*F*_(1,62)_ = 47.9; *p* < 0.0001) and that IL-1R, TNFR, NF-κB1, NF-κB2, RelA, and cRel mRNA levels were related to brain pH (all *F*_(1,62)_ ≥ 4.9; all *p* ≤ 0.03). No other postmortem factors were found to affect mRNA levels (all *F* ≤ 3.9, all *p* ≥ 0.053), and none of these factors differed between diagnostic groups (Table 1). Consequently, the ANCOVA model we report includes mRNA level as the dependent variable, diagnostic group as the main effect, age as a covariate only for HIVEP2 mRNA levels, and brain pH as a covariate only for IL-1R, TNFR, NF-κB1, NF-κB2, RelA, and cRel mRNA levels. Because 10 different NF-κB-related mRNAs were quantified, the Holm simultaneous inference procedure was used to account for multiple comparisons^25^ as previously described^26^, and Bonferroni-Holm adjusted *p* values are reported for the primary statistical model. Each bipolar disorder subject was individually matched to a comparison subject to account for the parallel processing of tissue samples from a pair and to balance diagnostic groups for sex and age. Therefore, a second ANCOVA model was employed with subject pair as a blocking factor and including postmortem interval, brain pH, RIN, and storage time was also used. Because both ANCOVA models produced similar results, we only report the first model. Subsequent analyses of differences in mRNA levels were conducted using the first ANCOVA model between bipolar disorder subjects grouped according to the following: (1) presence or absence of psychotic features; (2) use of antipsychotic medications at time of death; (3) use of antidepressants at time of death; (4) use of benzodiazepines and/or valproic acid at time of death; (5) use of tobacco at time of death; (6) substance use disorder at time of death; and (7) suicide as manner of death (Supplemental Table S1). Pearson correlation coefficients (*r*) were calculated to assess the relationships between quantified mRNA levels. Using data from our prior study in schizophrenia subjects which found mean NF-κB2 mRNA expression ratios of 0.00202 ± 0.00047 in the unaffected comparison subjects, we estimated that the sample size of 35 bipolar disorder subjects in the current study provides statistical power of 0.80 and 0.95 to detect differences of 15 and 20%, respectively, in mRNA levels between groups.

## Results

### NF-κB family member transcript levels in the PFC in bipolar disorder

**Fig. 2:**
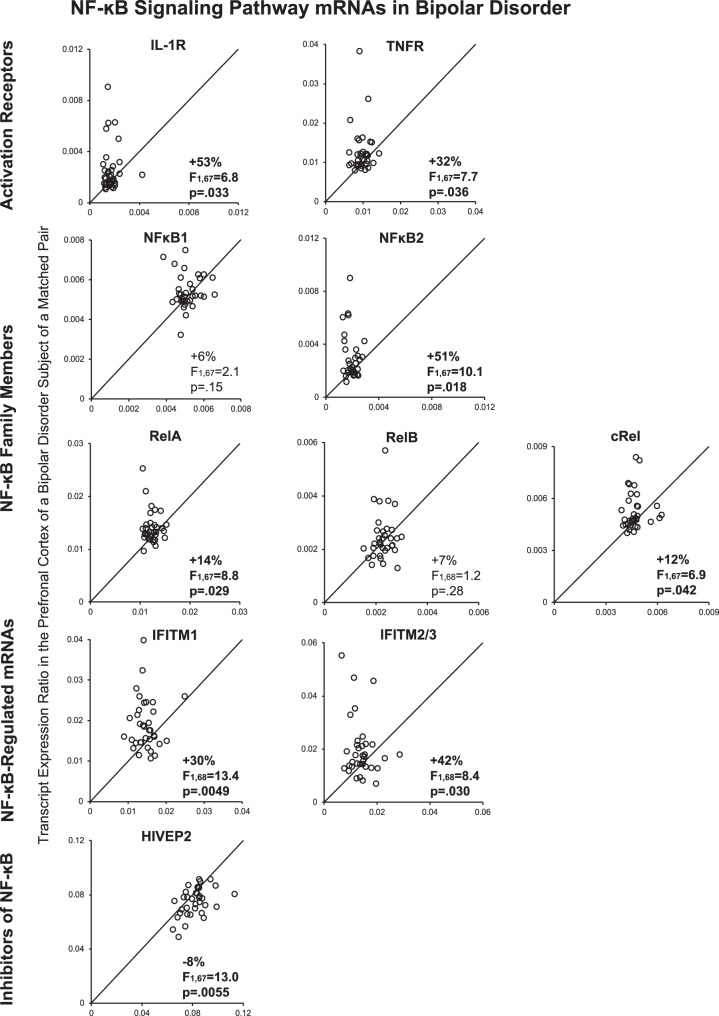
Transcript levels of NF-κB-related mRNAs in the PFC in bipolar disorder. Open circles represent mRNA levels for each bipolar disorder subject relative to the matched unaffected comparison subject. Data points to the left of the unity line indicate higher mRNA levels in the bipolar disorder subject relative to the comparison subject. Percent difference in diagnostic group means and primary statistical analysis results with Bonferroni-Holm adjusted *p* values are provided for each transcript. Mean ± standard deviation mRNA levels for unaffected comparison subjects and bipolar disorder subjects, respectively, are: IL-1R (0.0017 ± 0.00057 and 0.00259 ± 0.0018); TNFR (0.00962 ± 0.0018 and 0.0127 ± 0.0058); NF-κB1 (0.0051 ± 0.00058 and 0.0054 ± 0.00085); NF-κB2 (0.00196 ± 0.00041 and 0.00296 ± 0.0017); RelA (0.0123 ± 0.0013 and 0.0141 ± 0.0030); RelB (0.00228 ± 0.00034 and 0.00245 ± 0.00087); cRel (0.00468 ± 0.00054 and 0.00526 ± 0.0011); IFITM1 (0.0146 ± 0.0029 and 0.0190 ± 0.0064); IFITM2/3 (0.014 ± 0.0044 and 0.0199 ± 0.011); and HIVEP2 (0.0817 ± 0.010 and 0.075 ± 0.010).

We first determined whether mRNA levels for family members of the NF-κB transcriptional complex, including NF-κB1, NF-κB2, and other members of the NF-κB family that contain Rel homology domains including RelA, RelB, and cRel, were elevated in the PFC of bipolar disorder subjects. Bipolar disorder subjects had higher mean mRNA levels for NF-κB2 (+51%; *F*_(1,67)_ = 10.1, *p* = 0.018; Fig. 2), RelA (+14%; *F*_(1,67)_ = 8.8, *p* = 0.029), and cRel (+12%; *F*_(1,67)_ = 6.9, *p* = 0.042), but not NF-κB1 (+6%; *F*_(1,67)_ = 2.1, *p* = 0.15) or RelB (+7%; *F*_(1,68)_ = 1.2, *p* = 0.28), relative to unaffected comparison subjects. NF-κB2, RelA, and cRel mRNA levels were higher in 71%, 69 and 71%, respectively, of bipolar disorder subjects relative to their matched comparison subjects. Furthermore, we found NF-κB2 mRNA levels were correlated with mRNA levels for RelA (*r* = 0.83, *p* < 0.00001) and cRel (*r* = 0.74, *p* < 0.00001) across all subjects, as well as in either bipolar disorder subjects alone (all *r* ≥ 0.75, all *p* < 0.00001) or comparison subjects alone (all *r* ≥ 0.45, all *p* < 0.007; Table 2). These associations are consistent with a higher rate of formation of NF-κB heterodimers, which are required for activation of the NF-κB transcriptional complex^12^.

**Table 2:** Correlations between NF-κB-related mRNAs in human PFC.

NF-κB marker	TNFR	NF-κB1	NF-κB2	RelA	RelB	cRel	IFITM1	IFITM2/3	HIVEP2
IL-1R									
All	**0.88**	**0.61**	**0.83**	**0.86**	**0.48**	**0.78**	**0.76**	**0.83**	**−0.30**
Bipolar	**0.89**	**0.64**	**0.82**	**0.89**	**0.53**	**0.79**	**0.80**	**0.85**	−0.26
Unaffected	**0.71**	**0.46**	**0.58**	**0.56**	−0.08	**0.58**	0.24	**0.52**	−0.19
TNFR									
All	–	**0.56**	**0.84**	**0.88**	**0.59**	**0.73**	0.77	**0.79**	**−0.34**
Bipolar	–	**0.58**	**0.83**	**0.89**	**0.65**	**0.73**	0.79	**0.81**	−0.32
Unaffected	–	**0.49**	**0.76**	0.70	0.09	**0.55**	**0.38**	**0.41**	−0.20
NF-κB1									
All	–	–	**0.67**	0.75	**0.42**	**0.76**	**0.49**	0.48	−0.15
Bipolar	–	–	**0.75**	0.76	**0.48**	**0.79**	**0.52**	0.54	−0.12
Unaffected	–	–	**0.45**	0.76	0.17	**0.67**	**0.29**	0.19	−0.04
NF-κB2									
All	–	–	–	**0.83**	0.70	**0.74**	**0.64**	0.72	−0.26
Bipolar	–	–	–	**0.82**	0.77	**0.75**	**0.60**	0.71	−0.18
Unaffected	–	–	–	0.72	0.20	**0.45**	**0.43**	0.44	−0.23
RelA									
All	–	–	–	–	0.47	**0.76**	**0.78**	0.76	−0.24
Bipolar	–	–	–	–	0.52	**0.75**	**0.81**	0.79	−0.18
Unaffected	–	–	–	–	0.12	**0.61**	**0.40**	0.35	−0.09
RelB									
All	–	–	–	–	–	**0.45**	**0.30**	0.35	0.16
Bipolar	–	–	–	–	–	**0.50**	0.33	0.41	0.12
Unaffected	–	–	–	–	–	0.11	−0.08	−0.17	0.54
cRel									
All	–	–	–	–	–	–	**0.53**	0.60	−0.16
Bipolar	–	–	–	–	–	–	**0.52**	0.61	−0.20
Unaffected	–	–	–	–	–	–	0.23	0.29	0.21
IFITM1									
All	–	–	–	–	–	–	–	0.76	−0.40
Bipolar	–	–	–	–	–	–	–	0.81	−0.41
Unaffected	–	–	–	–	–	–	–	0.27	−0.14
IFITM2/3									
All	–	–	–	–	–	–	–	–	−0.33
Bipolar	–	–	–	–	–	–	–	–	−0.28
Unaffected	–	–	–	–	–	–	–	–	−0.26

Values are Pearson correlation coefficients (*r*) for all subjects (*n* = 70), bipolar disorder subjects only (*n* = 35), and unaffected comparison subjects only (*n* = 35). Bold font indicates *p* < 0.05.

### NF-κB activation receptor transcript levels in the PFC in bipolar disorder

Next, we determined whether receptors that initiate NF-κB transcriptional activity (i.e. interleukin-1 receptor (IL-1R) and tumor necrosis factor receptor (TNFR))^12^ are also altered in bipolar disorder (Fig. 1). Mean mRNA levels were higher for IL-1R (+53%; *F*_(1,67)_ = 6.8, *p* = 0.033) and TNFR (+32%; *F*_(1,67)_ = 7.7, *p* = 0.036) in the PFC of bipolar disorder subjects relative to comparison subjects (Fig. 2). In addition, mRNA levels for these receptors were higher in 60% (IL-1R) and 77% (TNFR) of bipolar disorder subjects relative to their matched comparison subject. Furthermore, both IL-1R and TNFR mRNA levels were each correlated with mRNA levels for NF-κB2, RelA, and cRel across all subjects (all *r* ≥ 0.56, all *p* < 0.00001), in bipolar disorder subjects alone (all *r* ≥ 0.73, all *p* < 0.00001), and in comparison subjects alone (all *r* ≥ 0.45, all *p* ≤ 0.001; Table 2). Elevated levels of these receptors are consistent with increased NF-κB transcriptional activity in bipolar disorder.

### Levels of NF-κB-regulated transcripts in the PFC in bipolar disorder

The NF-κB complex regulates the transcription of immune-related mRNAs, such as IFITMs (Fig. 1)^10^. In bipolar disorder subjects, transcript levels were higher for IFITM1 (+30%; *F*_(1,68)_=13.4, *p* = 0.0049) and IFITM2/3 (+42%; *F*_(1,68)_ = 8.4, *p* = 0.030) relative to comparison subjects (Fig. 2). In addition, mRNA levels were higher in 71% (IFITM1) and 71% (IFITM2/3) of bipolar disorder subjects relative to their matched comparison subjects. Furthermore, IFITM1 mRNA levels were correlated with mRNA levels for IL-1R, TNFR, NF-κB2, and RelA across all subjects (all *r* ≥ 0.41, all *p* ≤ 0.004) and in bipolar disorder subjects alone (all *r* ≥ 0.60, all *p* ≤ 0.0001). In comparison subjects, IFITM1 mRNA levels were correlated with TNFR, NF-κB2, and RelA (all *r* ≥ 0.38, all *p* ≤ 0.026; Table 2) but not with IL-1R or cRel (all *r* ≤ 0.24, all *p* ≥ 0.16). Similarly, IFITM2/3 mRNA levels were correlated with mRNA levels for IL-1R, TNFR, NF-κB2, RelA, and cRel across all subjects (all *r* ≥ 0.48, all *p* ≤ 0.00002), in bipolar disorder subjects alone (all *r* ≥ 0.44, all *p* ≤ 0.008), and in comparison subjects alone (all *r* ≥ 0.35, all *p* ≤ 0.038) except for cRel (*r* = 0.29, *p* = 0.097). Findings of elevated NF-κB family members, initiation receptors, and NF-κB-regulated transcripts in bipolar disorder subjects, and strong correlations between these NF-κB-related mRNA levels, together suggest that NF-κB activity is elevated in the PFC in bipolar disorder.

### Inhibitors of NF-κB activity in the PFC in bipolar disorder

The NF-κB site-binding protein HIVEP2 inhibits NF-κB transcriptional activity (Fig. 1), and mice with Schnurri-2 (the mouse ortholog of HIVEP2) deficits have higher cortical levels of IFITM^27^. HIVEP2 mRNA levels were lower in bipolar disorder subjects (−8%; *F*_(1,67)_ = 13.0, *p* = 0.0055; Fig. 2). In addition, HIVEP2 mRNA levels were lower in 71% of bipolar disorder subjects relative to their matched comparison subject. Furthermore, HIVEP2 mRNA levels were negatively correlated with mRNA levels for IL-1R, TNFR, NF-κB2, RelA, IFITM1, and IFITM2/3 (all *r* ≤ −0.24, all *p* ≤ 0.044), but not with cRel mRNA(*r* = −0.16, *p* = 0.18), across all subjects, but not in bipolar disorder subjects or comparison subjects alone (all *r* ≤ |0.32|, all *p* ≥ 0.065).

### Relationship between expression levels of NF-κB-related mRNAs and bipolar diagnosis, psychosis, and co-morbid factors

We did not see differences in mRNA levels for any measured NF-κB-related marker between bipolar disorder subjects with psychotic features (*n* = 11) relative to bipolar disorder subjects without psychotic features (*n* = 24; all *F* ≤ 3.3, all *p* ≥ 0.08). We also did not see differences in mRNA levels between subjects diagnosed with bipolar I disorder (*n* = 21) relative to subjects diagnosed with another bipolar disorder (*n* = 14; i.e. bipolar II disorder (*n* = 8) or bipolar disorder not otherwise specified (*n* = 6)) for any NF-κB-related marker (all *F* ≤ 2.5, all *p* ≥ 0.12), with the exception of HIVEP2 (+11% in bipolar I disorder subjects; *F*_(1,32)_ = 7.1, *p* = 0.012).

We also found that NF-κB-related mRNAs did not differ between bipolar disorder subjects as a function of treatment with antipsychotics (all *F* ≤ 3.1, all *p* ≥ 0.09; Supplemental Table S1), antidepressants (all *F* ≤ 3.4, all *p* ≥ 0.08), or benzodiazepines and/or valproic acid at time of death (all *F* ≤ 1.7, all *p* ≥ 0.20). Furthermore, only three bipolar disorder subjects in the present cohort were treated with lithium at time of death (Supplemental Table S1), and consequently the potential effect of lithium on the expression of NF-κB-related mRNAs was not analyzed. NF-κB-related mRNA levels also did not differ in bipolar disorder subjects as a function of tobacco use (all *F* ≤ 4.2, all *p* > 0.05; Supplemental Table S1) or substance use disorders at time of death (all *F* ≤ 3.8, all *p* ≥ 0.06). We found that NF-κB-related mRNA levels did not differ in bipolar disorder subjects as a function of suicide as manner of death (all *F* ≤ 1.6, all *p* > 0.21; Supplemental Table S1) with the exception of cRel mRNA levels which were 8% higher in bipolar disorder subjects who died by suicide relative to bipolar disorder subjects with natural or accidental manners of death (*F*_1,32_ = 5.3, *p* = 0.028). Finally, duration of illness, which was available for 29 of the 35 bipolar disorder subjects in this study (Supplemental Table S1), was not correlated with expression levels of any NF-κB-related mRNA species (all *r* < |0.33|, all *p* > 0.08) with the exception of NF-κB2 (*r* = −0.38, *p* = 0.044).

## Discussion

In this study, we investigated NF-κB signaling in the PFC of bipolar disorder subjects by quantifying mRNA levels for NF-κB family members (NF-κB1, NF-κB2, RelA, RelB, and cRel), receptors that initiate NF-κB signaling (IL-1R, TNFR), NF-κB transcriptional activity-regulated products (IFITMs), and an NF-κB inhibitor (HIVEP2). First, we found that bipolar disorder subjects have higher mRNA levels for three NF-κB family members, NF-κB2, RelA, and cRel, relative to unaffected comparison subjects. Transcript levels for NF-κB2, RelA, and cRel were strongly correlated with each other, which suggests the presence of higher levels of functional NF-κB heterodimers in the same bipolar disorder subjects. Second, transcript levels for receptors that initiate NF-κB signaling were also markedly higher in bipolar disorder subjects, and IL-1R and TNFR mRNA levels were strongly correlated with mRNA levels for NF-κB family members. Third, we found higher mRNA levels for NF-κB transcriptional activity-regulated gene products, IFITMs, and transcript levels for IFITMs were also correlated with NF-κB activation receptors and family members. Fourth, mRNA levels for these NF-κB-related markers were each elevated in most (60–77%) bipolar disorder subjects relative to their matched comparison subjects. Finally, in contrast, mRNA levels for HIVEP2, an NF-κB site-binding protein that inhibits NF-κB transcriptional activity, were lower in bipolar disorder subjects and were inversely correlated with mRNA levels for the other NF-κB-related markers. However, the difference in group means for HIVEP2 was small (−8%) and of uncertain significance. In summary, the majority of bipolar disorder subjects have (1) elevated mRNA levels for NF-κB family members, initiation receptors, and NF-κB-regulated transcripts, (2) lower mRNA levels for an NF-κB inhibitor, (3) positive correlations between NF-κB-related markers, and (4) negative correlations between these NF-κB-related markers and an NF-κB inhibitor. Taken together, these findings are consistent with the presence of elevated NF-κB transcriptional activity in the PFC in bipolar disorder (Fig. 1), which may contribute to cortical immune activation in the disorder.

Next, we investigated the potential effects that co-morbid variables, including psychotropic medications, substance use disorders, tobacco use, immune/inflammation-related illnesses, and suicide, may have on NF-κB-related mRNA levels in bipolar disorder. Concerning psychotropic medications, we report that transcript levels for NF-κB-related markers did not differ between bipolar subjects who were treated with antipsychotics, antidepressants, and benzodiazepines and/or valproic acid at time of death and bipolar disorder subjects who were not treated with these medications at time of death. In prior studies, we similarly reported that NF-κB-related mRNA levels did not differ in schizophrenia subjects as a function of treatment with these medications at time of death^6,13,14^. We also previously reported that chronic (~2 year) exposure to antipsychotic medications in otherwise healthy monkeys did not affect NF-κB-related mRNA levels in the PFC^13,14^. However, interpretations of these results must take into account the possibility that exposure to different classes of psychotropic medications earlier in the lives of individuals with bipolar disorder may potentially impact the expression of NF-κB-related mRNAs in the PFC later in life. Furthermore, antipsychotic medications may have different effects in the brains of individuals with bipolar disorder than in otherwise healthy monkeys, and the effects of mood stabilizers were not assessed in this cohort of monkeys. While we also did not find differences in NF-κB-related mRNA levels in bipolar disorder subjects as a function of tobacco use or a diagnosis of substance use disorder at time of death, we similarly cannot exclude the possibility that tobacco use or illicit substance abuse earlier in life may have long-lasting effects on NF-κB-related mRNA levels quantified at the time of death. In addition, our subjects come from a community-based population (i.e. mostly middle-aged individuals with sudden, unexpected deaths and subsequent autopsies), had short agonal states (<24 h), and did not die from immune/inflammation-related causes of death. However, it remains possible that individuals with bipolar disorder may have had inflammatory diseases during their lifetime and the potential long-lasting effects of inflammatory disease earlier in life (if present) on the expression of NF-κB-related mRNAs in the PFC later in life is unknown. Finally, we found that NF-κB-related mRNA levels did not differ in bipolar disorder subjects as a function of suicide as manner of death relative to bipolar disorder subjects with natural or accidental manners of death with the exception of cRel mRNA levels which were 8% higher in bipolar disorder subjects who died by suicide.

Some limitations to the interpretability of the findings of this study are important to consider. First, while this study reports robust increases in mRNA levels for NF-κB-related markers and suggests the presence of enhanced NF-κB transcriptional activity in the PFC in bipolar disorder, additional studies will be needed to determine the extent to which these alterations in mRNA levels may result in changes in protein levels of NF-κB-related markers. For example, Rao et al.^28^ reported higher protein levels for IL-1R and NF-κB2 using western blot assays in the frontal cortex of a small cohort of bipolar disorder subjects (*n* = 10). Second, the use of quantitative PCR in PFC gray matter samples does not inform the individual cell populations that express elevated transcript levels for NF-κB-related markers in bipolar disorder. Previous studies in human brain tissue have reported that NF-κB family members and activation receptors are preferentially expressed in microglia, and to a lesser extent in astrocytes, but not in neurons or oligodendrocytes^13,29,30^ while IFITMs are expressed in microglia, astrocytes^29,30^ and also endothelial cells^6^. Therefore, additional studies are needed to determine whether elevated NF-κB activity influences microglia and astrocytes and their role in the regulation of cortical circuitry^31^. Finally, it is unlikely that potential residual white blood cell contamination in our tissue samples impacted the results of this study because we previously reported that multiple specific markers for different classes of white blood cells are not expressed at quantifiable levels in our gray matter homogenates.

Our findings of elevated NF-κB-related mRNA levels in the PFC in bipolar disorder are remarkably similar to our previous study of these transcripts in the PFC of subjects with schizophrenia. We previously found elevated mRNA levels for the same NF-κB activation receptors (IL-1R, TNFR) and family members (NF-κB2, RelA, cRel) and IFITMs, and lower levels of an NF-κB inhibitor (HIVEP2) in the PFC in schizophrenia^6,13,14^. There was one exception: NF-κB1 mRNA levels were significantly elevated (+18%) in the PFC in schizophrenia but were not significantly elevated (+6%) in the PFC in bipolar disorder. Furthermore, similar majorities of schizophrenia (60–81%) and bipolar disorder subjects (60–77%) had elevated mRNA levels for each quantified NF-κB-related marker relative to the matched unaffected comparison subject. This study also found that elevated NF-κB-related mRNA levels are present in bipolar disorder regardless of the presence or absence of psychosis. However, it should be noted that potential effects of the presence of psychosis may be difficult to detect because only 11 subjects had bipolar disorder with psychotic features in this study. Elevated NF-κB-related mRNA levels also do not appear to be attributable to disease chronicity because NF-κB-related mRNA levels were not correlated with duration of illness.

The similarities in these findings may indicate shared pathophysiological processes in bipolar disorder and schizophrenia. For example, microglia, which preferentially express NF-κB family members and activation receptors, are involved in the phagocytosis of dendritic spines on pyramidal neurons^32–39^. Spines, dendritic protrusions that receive most of the excitatory input to pyramidal neurons, are critical mediators of the cognitive functions^40,41^ that are impaired in bipolar disorder and schizophrenia^42,43^. The density of dendritic spines has been reported to be lower in deep layer 3 of the PFC in both bipolar disorder and schizophrenia^44–49^, and PFC layer 3 has been reported to subserve cognitive processes^50,51^ affected in these illnesses. While speculative, these findings suggest the possibility that enhanced NF-κB signaling may contribute to activation of microglia and possibly enhanced phagocytic activity of dendritic spines which may contribute to the presence of dendritic spine deficits in both bipolar disorder and schizophrenia. However, additional studies of the molecular capacity of microglia to excessively digest spines in these disorders are needed to further investigate this potential pathophysiological process. In summary, these findings indicate that cortical immune activation is a core pathophysiological feature shared between bipolar disorder and schizophrenia.

## Supplementary information

Supplemental Table S1

Supplemental Table S2

## References

[1] Zanelli J (2010). Specific and generalized neuropsychological deficits: a comparison of patients with various first-episode psychosis presentations. Am. J. Psychiatry.

[2] International Schizophrenia Consortium (2009). Common polygenic variation contributes to risk of schizophrenia and bipolar disorder. Nature.

[3] Ripke S (2013). Genome-wide association analysis identifies 13 new risk loci for schizophrenia. Nat. Genet..

[4] Calabro M, Drago A, Sidoti A, Serretti A, Crisafulli C (2015). Genes involved in pruning and inflammation are enriched in a large mega-sample of patients affected by Schizophrenia and Bipolar Disorder and controls. Psychiatry Res..

[5] Williams HJ (2011). Most genome-wide significant susceptibility loci for schizophrenia and bipolar disorder reported to date cross-traditional diagnostic boundaries. Hum. Mol. Genet..

[6] Siegel BI, Sengupta EJ, Edelson JR, Lewis DA, Volk DW (2014). Elevated viral restriction factor levels in cortical blood vessels in schizophrenia. Biol. Psychiatry.

[7] Saetre P (2007). Inflammation-related genes up-regulated in schizophrenia brains. BMC Psychiatry.

[8] Arion D, Unger T, Lewis DA, Levitt P, Mirnics K (2007). Molecular evidence for increased expression of genes related to immune and chaperone function in the prefrontal cortex in schizophrenia. Biol. Psychiatry.

[9] Cai HQ (2020). Increased macrophages and changed brain endothelial cell gene expression in the frontal cortex of people with schizophrenia displaying inflammation. Mol. Psychiatry.

[10] Diamond MS, Farzan M (2013). The broad-spectrum antiviral functions of IFIT and IFITM proteins. Nat. Rev. Immunol..

[11] Tornatore L, Thotakura AK, Bennett J, Moretti M, Franzoso G (2012). The nuclear factor kappa B signaling pathway: integrating metabolism with inflammation. Trends Cell Biol..

[12] Hoesel B, Schmid JA (2013). The complexity of NF-kappaB signaling in inflammation and cancer. Mol. Cancer.

[13] Volk DW, Moroco AE, Roman KM, Edelson JR, Lewis DA (2019). The role of the nuclear factor-kappaB transcriptional complex in cortical immune activation in schizophrenia. Biol. Psychiatry.

[14] Volk DW (2015). Molecular mechanisms and timing of cortical immune activation in schizophrenia. Am. J. Psychiatry.

[15] Fillman SG (2013). Increased inflammatory markers identified in the dorsolateral prefrontal cortex of individuals with schizophrenia. Mol. Psychiatry.

[16] American Psychiatric Association. *DSM-IV. Diagnostic and Statistical Manual of Mental Disorders* 4th edn, Vol. 4 (American Psychiatric Association, 1994).

[17] Volk DW, Eggan SM, Lewis DA (2010). Alterations in metabotropic glutamate receptor 1alpha and regulator of G protein signaling 4 in the prefrontal cortex in schizophrenia. Am. J. Psychiatry.

[18] Volk DW, Austin MC, Pierri JN, Sampson AR, Lewis DA (2000). Decreased glutamic acid decarboxylase67 messenger RNA expression in a subset of prefrontal cortical gamma-aminobutyric acid neurons in subjects with schizophrenia. Arch. Gen. Psychiatry.

[19] Volk DW, Radchenkova PV, Walker EM, Sengupta EJ, Lewis DA (2011). Cortical opioid markers in schizophrenia and across postnatal development. Cereb. Cortex.

[20] Volk DW, Siegel BI, Verrico CD, Lewis DA (2013). Endocannabinoid metabolism in the prefrontal cortex in schizophrenia. Schizophrenia Res..

[21] Volk DW, Edelson JR, Lewis DA (2014). Cortical inhibitory neuron disturbances in schizophrenia: role of the ontogenetic transcription factor Lhx6. Schizophr. Bull..

[22] Volk DW, Chitrapu A, Edelson JR, Lewis DA (2015). Chemokine receptors and cortical interneuron dysfunction in schizophrenia. Schizophr. Res..

[23] Vandesompele J (2002). Accurate normalization of real-time quantitative RT-PCR data by geometric averaging of multiple internal control genes. Genome Biol..

[24] Volk DW, Radchenkova PV, Walker EM, Sengupta EJ, Lewis DA (2012). Cortical opioid markers in schizophrenia and across postnatal development. Cereb. Cortex.

[25] Holm S (1979). A simple sequentially rejective multiple test procedure. Scand. J. Stat..

[26] Volk DW, Austin MC, Pierri JN, Sampson AR, Lewis DA (2000). Decreased glutamic acid decarboxylase67 messenger RNA expression in a subset of prefrontal cortical gamma-aminobutyric acid neurons in subjects with schizophrenia. Arch. Gen. Psychiatry.

[27] Takao K (2013). Deficiency of schnurri-2, an MHC enhancer binding protein, induces mild chronic inflammation in the brain and confers molecular, neuronal, and behavioral phenotypes related to schizophrenia. Neuropsychopharmacology.

[28] Rao JS, Harry GJ, Rapoport SI, Kim HW (2010). Increased excitotoxicity and neuroinflammatory markers in postmortem frontal cortex from bipolar disorder patients. Mol. Psychiatry.

[29] Bennett ML (2016). New tools for studying microglia in the mouse and human CNS. Proc. Natl Acad. Sci. USA.

[30] Zhang Y (2016). Purification and characterization of progenitor and mature human astrocytes reveals transcriptional and functional differences with mouse. Neuron.

[31] Volk DW (2016). Role of microglia disturbances and immune-related marker abnormalities in cortical circuitry dysfunction in schizophrenia. Neurobiol. Dis..

[32] Tremblay ME, Lowery RL, Majewska AK (2010). Microglial interactions with synapses are modulated by visual experience. PLoS Biol..

[33] Schafer DP (2012). Microglia sculpt postnatal neural circuits in an activity and complement-dependent manner. Neuron.

[34] Sekar A (2016). Schizophrenia risk from complex variation of complement component 4. Nature.

[35] Stevens B (2007). The classical complement cascade mediates CNS synapse elimination. Cell.

[36] Paolicelli RC (2011). Synaptic pruning by microglia is necessary for normal brain development. Science.

[37] Sellgren CM (2017). Patient-specific models of microglia-mediated engulfment of synapses and neural progenitors. Mol. Psychiatry.

[38] Mallya AP, Wang HD, Lee HNR, Deutch AY (2019). Microglial pruning of synapses in the prefrontal cortex during adolescence. Cereb. Cortex.

[39] Filipello F (2018). The microglial innate immune receptor TREM2 is required for synapse elimination and normal brain connectivity. Immunity.

[40] Cahill ME (2009). Kalirin regulates cortical spine morphogenesis and disease-related behavioral phenotypes. Proc. Natl Acad. Sci. USA.

[41] Kim IH (2013). Disruption of Arp2/3 results in asymmetric structural plasticity of dendritic spines and progressive synaptic and behavioral abnormalities. J. Neurosci..

[42] Kahn RS, Keefe RS (2013). Schizophrenia is a cognitive illness: time for a change in focus. JAMA Psychiatry.

[43] Lesh TA, Niendam TA, Minzenberg MJ, Carter CS (2011). Cognitive control deficits in schizophrenia: mechanisms and meaning. Neuropsychopharmacology.

[44] Glantz LA, Lewis DA (2000). Decreased dendritic spine density on prefrontal cortical pyramidal neurons in schizophrenia. Arch. Gen. Psychiatry.

[45] Garey LJ (1998). Reduced dendritic spine density on cerebral cortical pyramidal neurons in schizophrenia. J. Neurol. Neurosurg. Psychiatry.

[46] Kolluri N, Sun Z, Sampson AR, Lewis DA (2005). Lamina-specific reductions in dendritic spine density in the prefrontal cortex of subjects with schizophrenia. Am. J. Psychiatry.

[47] Konopaske GT, Lange N, Coyle JT, Benes FM (2014). Prefrontal cortical dendritic spine pathology in schizophrenia and bipolar disorder. JAMA Psychiatry.

[48] MacDonald ML (2017). Selective loss of smaller spines in schizophrenia. Am. J. Psychiatry.

[49] Glausier JR, Lewis DA (2013). Dendritic spine pathology in schizophrenia. Neuroscience.

[50] Wang M (2013). NMDA receptors subserve persistent neuronal firing during working memory in dorsolateral prefrontal cortex. Neuron.

[51] Goldman-Rakic PS (1995). Cellular basis of working memory. Neuron.

