# Supplementary material for: Involvement of the nuclear factor-κB transcriptional complex in prefrontal cortex immune activation in bipolar disorder
Source: Transl Psychiatry. 2021 Jan 12;11:40. doi: 10.1038/s41398-020-01092-x (PMC7804457; doi:10.1038/s41398-020-01092-x)
Supplement: Supplementary file 1 — Supplemental Table S1 [file 41398_2020_1092_MOESM1_ESM.pdf]

Table S1. Demographic, postmortem, and clinical characteristics of bipolar disorder and unaffected comparison subjects

Unaffected Comparison Subjects									Bipolar Disorder Subjects														
Pair	Case	Sex/ Race	Age	PMI[a]	Storage Time[b]	RIN	pH	Cause of Death	Case	DSM IV diagnosis	Sex/ Race	Age	PMI[a]	Storage Time[b]	RIN	pH	Cause of Death	Illness Duration[c]	Tobacco ATOD	Anti-psychotic ATOD	Anti-depressant ATOD	Benzodiazepine/ Anticonvulsant ATOD	Lithium ATOD
1	10003	M/W	49	21.2	119	8.4	6.5	Trauma	1102	Bipolar NOS; ADR; ODC; OAC; OAR	M/W	50	12.1	129	8.3	6.7	ASCVD	32	Yes	No	Yes	Yes	No
2	1374	M/W	43	21.7	90	7.2	6.6	ASCVD	1121	Bipolar I +P; ADR	M/W	40	18.5	127	8.3	6.4	Pulmonary embolism	12	No	Yes	No	Yes	No
3	1282	F/W	39	24.5	106	7.5	6.8	ASCVD	957	Bipolar I	F/W	39	22.2	150	8.4	6.7	Suicide by drowning	3	Unknown	No	Yes	No	No
4	1298	M/W	48	24.5	103	7.9	6.5	ASCVD	886	Bipolar I; ADC	M/W	45	27.2	165	8.3	7.1	Suicide by gun shot	20	Yes	No	No	No	No
5	1047	M/W	43	13.8	137	9.0	6.6	ASCVD	1020	Bipolar I +P; ADC; ODC	M/W	42	12.5	141	8.5	6.7	Accidental combined drug overdose	20	Yes	No	Yes	Yes	No
6	795	M/W	68	11.8	182	8.2	6.8	Ruptured aortic aneurysm	1130	Bipolar I +P; AAR	M/W	65	8.9	126	8.0	6.7	Esophageal cancer	35	Unknown	Yes	Yes	Yes	No
7	1789	F/W	53	13.7	15	8.5	6.8	ASCVD	1048	Bipolar I +P	F/W	51	21.5	136	7.7	6.7	Suicide by asphyxiation	25	Yes	No	Yes	No	No
8	1324	M/W	43	22.3	97	7.3	6.7	Aortic dissection	697	Bipolar I +P	M/W	39	24.2	200	7.8	6.6	Suicide by incised wounds	10	Unknown	No	No	Yes	No
9	1444	M/W	46	22.0	77	8.4	6.3	Pulmonary embolism	1069	Bipolar I; AAR	M/W	48	18.1	133	8.1	6.9	ASCVD	28	No	No	No	No	No
10	1086	M/W	51	24.2	131	8.1	6.6	ASCVD	1244	Bipolar I +P; AAR; ODC; OAC	M/W	52	23.5	111	8.0	6.7	Accidental asphyxiation	33	Yes	Yes	Yes	Yes	No
11	1391	F/W	51	7.8	86	7.1	6.6	ASCVD	10004	Bipolar I; ODC	F/W	50	11.7	118	8.5	6.4	Accidental combined drug overdose	Unknown	Unknown	Yes	No	No	No
12	1196	F/W	36	14.5	119	8.2	6.4	Asphyxiation	1180	Bipolar I +P	F/W	28	22.3	121	7.5	6.3	Suicide by jump	12	No	No	Yes	No	No
13	1293	F/W	65	18.5	104	7.0	6.5	Trauma	10006	Bipolar I +P; ADR	F/W	55	17.5	117	8.1	6.4	Suicide by gun shot	23	Yes	No	No	No	No
14	1153	M/W	55	28.0	123	8.0	6.1	ASCVD	716	Bipolar I; ADC; OAC	M/W	58	27.7	196	8.3	6.8	Suicide by gun shot	Unknown	Unknown	No	No	No	No
15	789	M/W	22	20.1	184	7.8	6.8	Asphyxiation	1181	Bipolar I +P; ODC; OAR	M/W	28	27.4	121	8.0	6.2	Accidental morphine overdose	6	Yes	No	Yes	Yes	No
16	686	F/W	52	22.6	201	8.5	7.1	ASCVD	1328	Bipolar NOS	F/W	49	21.5	97	7.5	6.7	ASCVD	15	No	No	Yes	Yes	Yes
17	1247	F/W	58	22.7	111	8.4	6.4	ASCVD	1044	Bipolar I +P	F/W	56	24.5	137	7.1	6.1	ASCVD	17	No	Yes	Yes	Yes	No
18	1092	F/B	40	16.6	130	8.0	6.8	Mitral valve prolapse	984	Bipolar I +P	F/W	42	31.2	146	8.0	6.5	Accidental combined drug overdose	24	Yes	Yes	Yes	No	Yes
19	840	F/W	41	15.4	172	9.1	6.6	ASCVD	945	Bipolar I; AAC	F/W	43	31.9	152	7.2	6.7	Suicide by asphyxiation	14	Yes	Yes	Yes	Yes	No
20	1031	M/W	53	23.2	139	8.9	6.8	ASCVD	849	Bipolar II	M/W	52	22.1	171	7.0	7.3	Suicide by alprazolam overdose	23	No	No	No	Yes	No
21	841	M/W	70	21.2	172	7.2	7.2	Hypertrophic cardiomyopathy	982	Bipolar II; ADR	M/W	79	31.1	146	8.4	6.7	Accidental smoke inhalation	29	Yes	No	Yes	Yes	No
22	1394	M/W	45	17.3	85	7.3	6.6	ASCVD	1207	Bipolar NOS; ADC; ODC	M/W	48	18.0	117	8.1	6.4	ASCVD	Unknown	Unknown	Yes	Yes	Yes	No
23	1081	F/W	57	14.9	131	9.0	6.8	Chronic obstructive pulmonary disease	1449	Bipolar II	F/W	57	19.1	75	8.0	6.4	ASCVD	27	Yes	No	Yes	No	No
24	1598	M/W	50	23.8	48	7.8	6.9	Acute myocardial infarction	1473	Bipolar II; AAC	M/W	51	22.3	72	8.1	6.6	Accidental electrocution	Unknown	Unknown	No	Yes	No	No
25	1694	F/W	67	8.5	31	8.9	6.7	Acute myocarditis	1483	Bipolar II; ADC; OAC	F/W	56	5.8	70	8.3	6	Chronic alcoholism	31	No	No	No	No	No
26	1403	F/W	45	12.3	84	8.2	6.4	ASCVD	1537	Bipolar II; ADR	F/W	52	21.1	58	8.9	6.8	Bilateral pulmonary emboli	24	Yes	No	Yes	Yes	No
27	1770	M/W	52	28.2	19	8.2	6.8	Asphyxiation	1565	Bipolar NOS; ADC	M/W	45	27.6	53	8.3	6.8	Acute & chronic myocardial infarct	8	Yes	Yes	Yes	No	No
28	1605	F/W	21	23.9	47	8.2	6.9	Arrhythmogenic right ventricular dysplasia	1589	Bipolar NOS; ADC; ODC; OAC	F/W	25	27.6	50	7.1	6.1	Accidental combined drug overdose	6	Yes	Yes	Yes	No	No
29	1489	M/W	25	16.9	70	8.3	5.9	Hypoglycemia	1603	Bipolar I; ADC; ODC	M/W	31	12.0	47	7.6	6.8	Suicide by hanging	Unknown	Yes	No	Yes	No	No

Unaffected Comparison Subjects									Bipolar Disorder Subjects														
**Pair**	**Case**	**Sex/ Race**	**Age**	**PMI[a]**	**Storage Time[b]**	**RIN**	**pH**	**Cause of Death**	**Case**	**DSM IV diagnosis**	**Sex/ Race**	**Age**	**PMI[a]**	**Storage Time[b]**	**RIN**	**pH**	**Cause of Death**	**Illness Duration[c]**	**Tobacco ATOD**	**Anti-psychotic ATOD**	**Anti-depressant ATOD**	**Benzodiazepine/ Anticonvulsant ATOD**	**Lithium ATOD**
30	10019	F/B	41	19.3	112	7.6	6.6	Complications of postpartum cardiomyopathy	1632	Bipolar II; ODC	F/B	31	19.1	43	8.5	6.6	Undetermined	6	No	No	No	No	No
31	1482	M/W	25	20.2	70	9.1	6.6	ASCVD	10014	Bipolar NOS; ADC; ODC	M/W	24	23.1	115	7.8	6.1	Suicide by gun shot	Unknown	Yes	No	No	No	No
32	838	M/W	58	16.5	173	8.5	7	ASCVD	1461	Bipolar I; AAR	M/W	57	7.6	73	8.7	6.8	ASCVD	42	No	Yes	Yes	No	No
33	1637	M/W	46	16.6	42	8.2	6.9	ASCVD	1584	Bipolar I; ODC; ODR	M/W	46	16.6	50	7.6	6.8	Suicide by oxymorphone overdose	10	Yes	No	Yes	Yes	Yes
34	1783	F/W	23	15.9	16	8.5	6.8	Trauma	1573	Bipolar II	F/W	27	11.2	52	9.0	6.7	Status asthmaticus	5	No	No	No	No	No
35	1429	M/B	44	23.9	79	7.5	6.8	Dilated cardiomyopathy & cardiomegaly	1695	Bipolar I; ADR; ODC; OAR	M/W	33	29.1	31	7.7	6.8	Accidental combined drug overdose	19	Yes	Yes	No	Yes	No
		Mean	46.4	19.1	103.0	8.1	6.7					45.5	20.5	109.9	8.0	6.6			18Y/10N/ 7Unk	12Y/23N	22Y/13N	16Y/19N	3Y/32N
		SD	12.7	5.1	49.8	0.6	0.3					12.2	7.0	45.1	0.5	0.3							

[a] PMI, postmortem interval (hours); [b] Storage time (months) at -80C; [c] Illness duration (years); Other abbreviations: ASCVD, arteriosclerotic cardiovascular disease; MCA, middle cerebral artery; ATOD, at time of death; ADC, alcohol dependence, current at time of death; ADR, alcohol dependence, in remission at time of death; AAC, alcohol abuse, current at time of death; AAR, alcohol abuse, in remission at time of death; Bipolar I +P, bipolar I disorder with psychotic features; Bipolar NOS, bipolar disorder not otherwise specified; ODC, other substance dependence, current at time of death; ODR, other substance dependence, in remission at time of death; OAC, other substance abuse, current at time of death; OAR, other substance abuse, in remission at time of death; U, unknown; M, male; F, female; W, white; B, black
