# Supplementary material for: Involvement of the nuclear factor-κB transcriptional complex in prefrontal cortex immune activation in bipolar disorder
Source: Transl Psychiatry. 2021 Jan 12;11:40. doi: 10.1038/s41398-020-01092-x (PMC7804457; doi:10.1038/s41398-020-01092-x)
Supplement: Supplementary file 2 — Supplemental Table S2 [file 41398_2020_1092_MOESM2_ESM.pdf]

Supplemental Table S2: qPCR primer design

Gene	Species	Accession #	Amplicon Size (bp)	Position	Forward Primer (F)<br>Reverse Primer (R)
Interleukin-1 receptor (IL-1R)	Human	NM_000877	102	1789-1890	(F) GCTTGAGCTGGAGAAAATCC<br>(R) TGTGTAAAGTCCCCTGACCA
Tumor necrosis factor receptor superfamily member 1A (TNFR)	Human	NM_001065.3	117	625-741	(F) GAAATGGGTCAGGTGGAGAT<br>(R) GCAATTGAAGCACTGGAAAA
Nuclear factor-κB isoform 1 (NF-κB1)	Human	NM_003998	143	819-961	(F) CACTGTGAGGATGGGATCTG<br>(R) CCCCTTATACACGCCTCTGT
Nuclear factor-κB isoform 2 (NF-κB2)	Human	NM_001077494	122	960-1081	(F) TCTCGAATGGACAAGACAGC<br>(R) TGCCATCCATTCTCATCATC
RelA	Human	NM_021975.3	50	514-563	(F) ACCTGGAGCAGGCTATCAGT<br>(R) GAAGGGGTTGTTGTTGGTCT
RelB	Human	NM_006509.3	81	1048-1128	(F) GCCGAATTAACAAGGAAAGC<br>(R) TGTCCTCTTTCTGCACCTTG
cRel	Human	NM_002908.3	83	1472-1554	(F) ATGCCTACAGGGGTTTCAAG<br>(R) GAGGCATGATGTGACAATCC
Interferon-induced transmembrane protein 1 (IFITM1)	Human	NM_003641	82	576-657	(F) CAACCTTTGCACTCCACTGT<br>(R) GTATCTAGGGGCAGGACCAA
IFITM2/3*	Human	NM_006435 (IFITM2)<br>NM_021034 (IFITM3)	59	441-499 (IFITM2)<br>459-517 (IFITM3)	(F) CTGCTCATCATCATCCCAGT<br>(R) TGATGCCTCCTGATCTATC
HIVEP2	Human	NM_006734	148	1610-1757	(F) CGAGGCTTCTGACAAAATGA<br>(R) AGGGAGAGGAATCCCACTTT
Beta actin	Human	NM_001101	101	1146-1246	(F) GATGTGGATCAGCAAGCA<br>(R) AGAAAGGGTGTAACGCAACTA
Cyclophilin	Human	NM_021130	126	159-284	(F) GCAGACAAGGTCCCAAAG<br>(R) GAAGTCACCACCCTGACAAC
Glyceraldehyde-3-phosphate dehydrogenase (GAPDH)	Human	NM_002046	87	556-642	(F) TGCACCACCAACTGCTTAGC<br>(R) GGCATGGACTGTGGTCATGAG

* Due to the very high sequence homology between IFITM2 and IFITM3 in humans, this primer set was designed to target an mRNA region common to both IFITM2 and IFITM3 (termed IFITM2/3).
